# Supplementary material for: The impact of latent tuberculosis screening programmes for migrant populations in high income, low burden countries
Source: PLoS One. 2025 Nov 13;20(11):e0335904. doi: 10.1371/journal.pone.0335904 (PMC12614610; doi:10.1371/journal.pone.0335904)
Supplement: S1 Appendix — (DOCX) [file pone.0335904.s001.docx]

*S1 Appendix: Search strategy*

| Date of Search | 27 February 2024 |
| --- | --- |
| Bibliographic databases used | Medline, Embase, Scopus, Proquest Public Health |
| Grey literature sources used | Google search, Idox Knowledge Exchange, Policy Commons, WHO, OECD iLibrary, European Centre for Disease Prevention and Control, HSC Public Health Agency (Northern Ireland), GOV.UK, PHS, Public Health Wales |

**Database:** Ovid MEDLINE(R) ALL <1946 to February 26, 2024>

| # | Query |
| --- | --- |
| 1 | exp Tuberculosis/ |
| 2 | exp Mycobacterium tuberculosis/ |
| 3 | (tuberculos* or tb*).ti,ab,kf. |
| 4 | 1 or 2 or 3 |
| 5 | (latent or asymptomatic or inactive).ti,ab,kf. |
| 6 | 4 and 5 |
| 7 | exp Latent Tuberculosis/ |
| 8 | LTBI.ti,ab,kf. |
| 9 | 7 or 8 |
| 10 | 6 or 9 |
| 11 | exp Mass Screening/ |
| 12 | exp Tuberculin Test/ |
| 13 | exp Interferon-gamma Release Tests/ |
| 14 | (screen* or test* or diagnos* or "tuberculin skin test" or "interferon gamma release assay" or IGRA or TST).ti,ab,kf. |
| 15 | 11 or 12 or 13 or 14 |
| 16 | 10 and 15 |
| 17 | exp "Emigrants and Immigrants"/ |
| 18 | exp "Emigration and Immigration"/ |
| 19 | exp "Transients and Migrants"/ |
| 20 | (refugee* or "asylum seeker*" or migrat* or immigra* or emigrat*).ti,ab,kf. |
| 21 | 17 or 18 or 19 or 20 |
| 22 | 16 and 21 |
| 23 | (Australia or Australian or Austria or Austrian or Belgium or Belgian or Canada or Canadian or Denmark or Danish or Finland or Finnish or France or French or Germany or German or Hungary or Hungarian or Ireland or Irish or Israel or Israeli or Italy or Italian or Japan or Japanese or Netherlands or Dutch or "New Zealand" or Norway or Norwegian or Spain or Spanish or Sweden or Swedish or Switzerland or Swiss or "United Kingdom" or "Great Britain" or UK or Scotland or Scottish or Wales or Welsh or England or English or "United States of America" or "United States" or US or America).ti,ab,kf. |
| 24 | 22 and 23 |
| 25 | limit 24 to english language |
| 26 | limit 25 to yr="2000 -Current" |

**Database:** Embase <1974 to 2024 February 26>

| **#** | **Query** |
| --- | --- |
| 1 | exp tuberculosis/ |
| 2 | exp Mycobacterium tuberculosis/ |
| 3 | exp tuberculosis control/ |
| 4 | (tuberculos* or tb*).ti,ab,kf. |
| 5 | 1 or 2 or 3 or 4 |
| 6 | (latent or asymptomatic or inactive).ti,ab,kf. |
| 7 | 5 and 6 |
| 8 | exp latent tuberculosis/ |
| 9 | LTBI.ti,ab,kf. |
| 10 | 8 or 9 |
| 11 | 7 or 10 |
| 12 | exp mass screening/ |
| 13 | exp tuberculin test/ |
| 14 | exp interferon gamma release assay/ |
| 15 | exp screening/ |
| 16 | exp screening test/ |
| 17 | (screen* or test* or diagnos* or "tuberculin skin test" or "interferon gamma release assay" or IGRA or TST).ti,ab,kf. |
| 18 | 12 or 13 or 14 or 15 or 16 or 17 |
| 19 | exp immigrant/ |
| 20 | exp migration/ |
| 21 | exp immigration/ |
| 22 | exp migrant/ |
| 23 | (refugee* or "asylum seeker*" or migrat* or immigra* or emigrat*).ti,ab,kf. |
| 24 | 19 or 20 or 21 or 22 or 23 |
| 25 | 11 and 18 |
| 26 | 24 and 25 |
| 27 | (Australia or Australian or Austria or Austrian or Belgium or Belgian or Canada or Canadian or Denmark or Danish or Finland or Finnish or France or French or Germany or German or Hungary or Hungarian or Ireland or Irish or Israel or Israeli or Italy or Italian or Japan or Japanese or Netherlands or Dutch or "New Zealand" or Norway or Norwegian or Spain or Spanish or Sweden or Swedish or Switzerland or Swiss or "United Kingdom" or "Great Britain" or UK or Scotland or Scottish or Wales or Welsh or England or English or "United States of America" or "United States" or US or America).ti,ab,kf. |
| 28 | 26 and 27 |
| 29 | limit 28 to english language |
| 30 | limit 29 to yr="2000 -Current" |
| 31 | limit 30 to (article or "preprint (unpublished, non-peer reviewed)") |

**Database:** Scopus

( TITLE-ABS-KEY ( "latent tuberculos*" OR "latent tb" OR ltbi ) AND TITLE-ABS-KEY ( screen* OR test* OR diagnos* OR "tuberculin skin test" OR "interferon gamma release assay" OR IGRA OR TST) AND TITLE-ABS-KEY ( emigrant* OR refugee* or "asylum seeker*" or migrat* or immigra*) AND TITLE-ABS-KEY ( australia OR australian OR austria OR austrian OR belgium OR belgian OR canada OR canadian OR denmark OR danish OR finland OR finnish OR france OR french OR germany OR german OR hungary OR hungarian OR ireland OR irish OR israel OR israeli OR italy OR italian OR japan OR japanese OR netherlands OR dutch OR "New Zealand" OR norway OR norwegian OR spain OR spanish OR sweden OR swedish OR switzerland OR swiss OR "United Kingdom" OR "Great Britain" OR uk OR scotland OR scottish OR wales OR welsh OR england OR english OR "United States of America" OR "United States" OR us OR america ) ) AND PUBYEAR > 1999 AND ( LIMIT-TO ( LANGUAGE , "English" ) )

**Database:** Proquest Public Health

noft("latent tuberculos*" OR "latent tb" OR ltbi) AND noft(screen* OR test* OR diagnos* OR "tuberculin skin test" OR "interferon gamma release assay" OR IGRA OR TST) AND noft(emigrant* OR refugee* or "asylum seeker*" or migrat* or immigra*) AND (australia OR australian OR austria OR austrian OR belgium OR belgian OR canada OR canadian OR denmark OR danish OR finland OR finnish OR france OR french OR germany OR german OR hungary OR hungarian OR ireland OR irish OR israel OR israeli OR italy OR italian OR japan OR japanese OR netherlands OR dutch OR "New Zealand" OR norway OR norwegian OR spain OR spanish OR sweden OR swedish OR switzerland OR swiss OR "United Kingdom" OR "Great Britain" OR uk OR scotland OR scottish OR wales OR welsh OR england OR english OR "United States of America" OR "United States" OR us OR america)
