# Supplementary material for: The impact of latent tuberculosis screening programmes for migrant populations in high income, low burden countries
Source: PLoS One. 2025 Nov 13;20(11):e0335904. doi: 10.1371/journal.pone.0335904 (PMC12614610; doi:10.1371/journal.pone.0335904)
Supplement: S2 Table — (DOCX) [file pone.0335904.s002.docx]

*S2 Table: Summary of literature evaluating epidemiology of migrants eligible for or screened in a latent TB screening programmes.*

| Country | Model (author, year) | Population | Identification method | Test method used | Outcomes |
| --- | --- | --- | --- | --- | --- |
| Australia | Schools [(Sawka et al, 2019](https://onlinelibrary.wiley.com/doi/full/10.1111/resp.13491)) | Newly arrived school children from 'high risk' countries of origin | School system | TST | 17.9% of TSTs were positive. 15.9% of the students referred completed a course of IPT.10.7% of those who started treatment did not finish or were transferred. |
| Canada | New arrivals clinic [(Pépin et al. 2022)](https://journals.plos.org/plosone/article?id=10.1371/journal.pone.0267781) | Adult & child asylum seekers or refugees (status obtained prior to landing) | Clients presenting to Sherbrook refugee clinic | Predominantly TST | NNS: 95.1. NNT: 11.9. 8.6% diagnosed with latent TB. 85% completed treatment.  Delivered as part of an integrated migrant health package. |
|  | [(Rennert-May et al. 2016)](https://www.ncbi.nlm.nih.gov/pmc/articles/PMC4904499/) | Newly entered government sponsored refugees of all ages, from all countries of origin | Referred on arrival by reception house | TST +/- IGRA | 949 tests offered. 746 TSTs were read and 265 were positive. 203 went on to have IGRAs, of which 110 were positive. 151 people were diagnosed with LTBI. 141/147 people offered treatment accepted it, with 103 completing treatment. |
|  | Schools [(Brassard et al. 2006)](https://pubmed.ncbi.nlm.nih.gov/16452324/) | Newly arrived children aged 4-18 | Annual TB clinic in schools run by Montreal Children's Hospital at welcoming classes for newly arrived immigrant children. Cascade screening of family members | TST | 3710 TSTs offered, 2524 completed. 542/2524 (21% positive). 484 presented at TB clinic. 441 diagnosed with TLBI. 342 started treatment with 78 lost to follow up. Family members of TST positive children were tested in family appointments: 599 investigated. 555 with results, 38% of these found to be TST +ve. |
|  | [(Minodier et al. 2010)](https://pubmed.ncbi.nlm.nih.gov/20701894/) | Immigrant school children | School nurses informed children and parents by mail. A TB trained nurse visited schools | TST | 4375 tests offered, 3602 TSTs completed, 3401 read. 777/3401 positive. 334/573 starting treatment completed it. |
| England | Primary care [(Loutet et al. 2018)](https://pubmed.ncbi.nlm.nih.gov/29326327/) | Migrant populations born or lived in countries of high incidence (>150/100,000) or SSA and entered the UK in the last 5 years. | GP registration | IGRA | 5591 tests offered, 2269 IGRAs. 719 positive. 11 people were diagnosed with active TB. 449 people started treatment. 103 people did not attend GP following positive IGRA. IGRA positivity was associated with increasing age, male gender, >36 years, born in SSA and diabetes. Found a big variation in screening uptake between GP surgeries (0-88%). |
|  | [(Panchal et al. 2014)](https://pubmed.ncbi.nlm.nih.gov/24253833/) | New migrants of all ages registering with a GP from a previous abroad address. | GP Flag-4 coded registration (indicates last address was abroad) | Not stated | 28438 (48%) of Flag 4 registrations were from countries with an incidence >150/100k. Median time of GP registration was 181 days from GP entry, but 619 days from people arriving from high incidence countries. 29.2% of foreign-born TB could have been preventable with screening. >50% of foreign-born TB was not preventable with GP based screening, due to failure to register with GP. Delay to Flag-4 registration was nearly 3x longer in immigrants progressing to active TB. |
|  | [(Berrocal-Almanza et al. 2022)](https://www.ncbi.nlm.nih.gov/pmc/articles/PMC8967722/) | Migrants aged 15-35 years arrived in the UK within 5 years from a country of TB incidence >150/100,000 population. | National retrospective study | IGRA | Of 330829 not tested for LTBI, 1280 TB cases. 37268 tested: In IGRA-ve 1280 TB out of 29829. 135 TB cases out of 6640 IGRA+ve. 1740 treated: 7 TB cases. 4900 untreated: 128 cases. |
|  | [(Berrocal-Almanza et al. 2019)](https://pubmed.ncbi.nlm.nih.gov/31471131/) | Migrants aged 16-35 years arrived in the UK within 5 years from a country of TB incidence >150/100,000 population, identified through GP registration. | GP Flag-4 coded registration | Not stated | 1% of the migrants registered in primary care in the UK were screened. Individuals more likely to be screened were ages 16-25 years, those with settlements and dependents or via family reunion. 2451 people were tested and 17.1% diagnosed with LTBI. 17,938 migrants migrated on to Scotland or Northern Ireland over the 3-year study period. |
|  | [(Public Health England, 2021](https://assets.publishing.service.gov.uk/media/6046616ad3bf7f1d1420700c/LTBI-testing-and-treatment_2019_to_2020.pdf)) | Migrants aged 16-35 years arrived in the UK within 5 years from a country of birth or lived > 6 months in a country of TB incidence >150/100,000 population. | Report on the national screening programme | IGRA | 22,221 tests were received in 2020 and the positivity rate has been decreasing 2015-2022. More women tested than men, but men have higher positivity rates. 56% of those tested are from India and Pakistan. Treatment completion is available for 45% of those with a positive test and completion rates of 75% of those who started treatment. Positivity rate has declined from 22% in 2015 to 2016 to 16% in 2019 to 2020. |
|  | [(Pareek et al. 2013)](https://pubmed.ncbi.nlm.nih.gov/22693179/) | Foreign-born migrants aged >16 years, entered within 5 years from a country of incidence >40/100,000 population or all countries + TB symptoms. | Via GP registrations | Stratified TST. | 321 tests were completed. There was an association between both age TB incidence in country of origin with screening positivity. |
|  | Screening setting not specified [(Zenner et al. 2017)](https://www.ncbi.nlm.nih.gov/pmc/articles/PMC5540676/) | New migrants <35 years through registration with GP or port of entry referral arriving from 'high incidence' countries of origin. | Primary care registration or referral from port of arrival after exclusion of active TB. | TST 1989-2001, IGRA 2009-2013 | 1820 tests were completed (1341 IGRA, 479 IGRAs). 366/1341 IGRAs were positive. 279/479 TSTS were positive. 243 people started treatment and 123 did not. Of the untreated LTBI +ve IGRA cohort, 13/124 patients developed active TB (10.6%). 1.7% (4/123) LTBI treated positive patients developed active TB. In the untreated LTBI cohort, 54% of cases happened >5 years after entry to UK. |
|  | TB centres [(Pareek et al. 2011)](https://pubmed.ncbi.nlm.nih.gov/21514236/) | Foreign-born migrants aged <16 years, entered within 5 years from countries of all incidences. Referred through port-of-entry screening systems, health-protection units, or after registration with primary-care services. | Referred for screening through port-of-entry, via GP, or by health protection units | IGRA | 245/1229 IGRAs were positive. Applying current (now-outdated) NICE guidelines would result in screening for 22% of individuals and identify 29% of LTBI. |
|  | Nurse-led screening clinic [(Gasmelseed et al. 2022)](https://thorax.bmj.com/content/77/Suppl_1/A166.2) | Migrants aged 16-35 years arrived in the UK within 5 years from a country of TB incidence >150/100,000 population, identified through GP registration. | NHS Flag 4 data, invited, unclear how invite sent. Funded by UKHSA | Not stated | 2525 invited for screened and 735 tests completed. 147/735 (20%) of people tested were diagnosed with LTBI and 7 with active TB. 15 patients were diagnosed with active TB in the clinic who had been invited to for screening but had not attended |
|  | Paediatric infectious diseases clinic [(Williams et al. 2020)](https://pubmed.ncbi.nlm.nih.gov/32094246/) | Unaccompanied asylum-seeking children, newly entered, | Mandatory referral by the receiving local authority, normally within 28 days | Not stated | 252 children tested, 23% were positive. |
| Germany | Unclear [(Thee et al. 2019)](https://www.ncbi.nlm.nih.gov/pmc/articles/PMC6528979/) | Unaccompanied refugees, under 18 years | Local authorities and charity organisations contacted and asked to inform the refugees they cared for. | IGRA | 970 tests offered, 301 IGRAs completed and 42 were positive. 38/48 were diagnosed with LTBI. Of the 38 people who started treatment, 29 finished (9 lost to follow up). |
|  | Reception centres [(Mueller-Hermelink et al. 2018)](https://www.ncbi.nlm.nih.gov/pmc/articles/PMC6205260/) | All asylum-seeking children aged 3-15 years. | Asylum seeker reception centre before moving on to temporary accommodation | Hamburg: TST. Bochum: <5 years TST, >5 years initially IGRA due to TST shortage | 66/968 (6.8%) of screened children had TB infection (58 LTBI, 8 active TB). LTBI prevalence was similar in children from high (Afghanistan) and low (Syria) incidence countries (8.7% vs 6.4%). Children under the age of 6 years were at higher risk of progression to active TB (19% vs 2% respectively, p=0,07). 7/8 children with active TB were asymptomatic at the time of diagnosis. The risk of developing TB in adults asylum seekers is higher in comparison to country of origin. The Balkan route for migration appeared to be a risk factor for TB. |
| Italy | Centralised TB screening centre for homeless migrants [(Barcellini et al. 2018)](https://pubmed.ncbi.nlm.nih.gov/30236183/) | All foreign-born migrants arriving at reception centres ages <36 years | Via reception centre | TST + confirmatory IGRA | 9486 migrants tested. 2666 had positive TSTs and attended for confirmatory IGRAs, of which 50.2% were positive. 72 people diagnosed with active TB. The odds of being QFT-positive in individuals from countries with a TB incidence <50/100 000 were 5.17 times higher in individuals from countries with a TB incidence of 51–150/100,000k and 3.96 times higher in individuals from countries with a TB incidence of 151–250 and approximately 8 times higher in individuals from countries of >250/100 000 |
|  | Specialist migrant clinic [(Carvalho et al. 2005)](https://pubmed.ncbi.nlm.nih.gov/15724725/) | Undocumented immigrants from countries of incidence >50/100,000k, entered recently with the intention to live or work in the region for at least six months, aged 18-35 years. | Consented for screening study if attended the medical clinic | TST | 649 TSTs were administered and 213 returned for reading of tests. 19% of all TSTs were positive. 55 people accepted LTBI treatment. Compared to a previous study at the same clinic with higher screening adherence, the population were younger and had a reduced duration of stay. |
|  | Specialist migrant clinic [(Bordin et al. 2022)](https://pubmed.ncbi.nlm.nih.gov/35574886/) | All asylum seekers residing in the region and evaluated by the service for migrants. |  | TST only if >15mm, confirmatory IGRA if TST 5-14mm | 2486 people were eligible and 2418 attended. 29% of people invited for screening were diagnosed with latent TB. 81% of people diagnosed accepted treatment and 70% completed it. The 30% who didn't complete treatment were due to drop out or transfer. |
|  | Specialist immigrant clinic [(Bonvicini et al. 2018)](https://www.ncbi.nlm.nih.gov/pmc/articles/PMC6339090/) | Irregular immigrants (those not entitled to a GP), >15 years |  | TST with confirmatory IGRA | 349/368 completed TSTs were read. Treatment was started in 20/28 patients diagnosed with LTBI, with 14 completing the course of treatment. The study comments that had they excluded immigrants from countries with a low TB incidence they would have excluded 48% of subjects and missed 80% of TB cases. |
|  | Reception centres [(Villa et al. 2019)](https://erj.ersjournals.com/content/54/4/1900896) | Asylum seekers residing in Milan's reception centres | Residing in reception centres in Milan | TST then confirmatory IGRA in adults. Children <5 years: TST only | 5324 people were tested (mostly young, male and from SSA). 69 (1.3%) were diagnosed with active TB and 863 (16.4%) with latent TB. 1339 people completed confirmatory IGRAs and 865 were positive (64.6%). |
|  | Reception centres [(Marrone et al. 2020](https://pubmed.ncbi.nlm.nih.gov/32947054/)) | Unaccompanied immigrant minors (<18 years) on arrival to reception centre | Recruited upon arrival to reception centre | TST or TST with (dependent on IGRA availability) | 834 TSTs were completed. Latent TB prevalence was 12% as defined by TST-positive, or 6.6% with double positivity TST/IGRA. Country of origin was a strong predictor of positivity, particularly SSA. Longer permanence was associated with higher positivity. |
|  | Reception centres [(Pontarelli et al. 2019)](https://www.sciencedirect.com/science/article/abs/pii/S1477893918303703) | Asylum seekers in accredited reception centres. | Retrospective analysis of registered asylum seekers screened for TB. | TST | 88.4% of 2904 registered asylum seekers were evaluated for TB. Active TB yield was 155/100,000 person-years. TST positivity was 36.6%. Of 843 LTBI treatment candidates, 413 completed screening. Treatment was prescribed to 190 of 397 eligible individuals. 91 people completed treatment. |
| Norway | Reception centres [(Harstad et al. 2010)](https://pubmed.ncbi.nlm.nih.gov/19914972/) | All asylum seekers, identified via reception centres. | Via National Reception Centre | TST. | 2237 TSTs. 57% of eligible participants with a positive TST had no contact with any local health authorities. Only 30 asylum seekers were treated for latent TB with treatment starting at a median 17 months from entering Norway. |
|  | National Reception Centre [(Harstad et al. 2009)](https://bmcpublichealth.biomedcentral.com/articles/10.1186/1471-2458-9-141) | All asylum seekers >15 years, identified via reception centres. | On arrival all asylum seekers are referred to the national reception centre for management of immediate medical needs and compulsory TB screening. | TST | 97.5% were tested. 46% had a positive TST. Only 16% of those with a strongly positive TST were reviewed by a specialist |
|  | Not specified [(Winje et al. 2019](https://pubmed.ncbi.nlm.nih.gov/30782706/)) | Analysis of migrants from the top 10 TB source countries according to Norwegian data | Retrospective analysis of databases including the Norwegian Surveillance System for Infectious Disease | IGRA | Overall NNT and NNS for the top 10 source countries in Norway. NNS and NNT were lowest for Somalia: 70-150 and 19-41 respectively. NNT was highest for immigrant from Pakistan and Thailand. There was a stronger correlation between NNT and Norwegian notification rate than NNT and WHO IR. |
| Sweden | Primary care [(Nederby Öhd et al. 2021)](https://erj.ersjournals.com/content/57/3/2002255) | Asylum seekers from countries of incidence (>100/100,000) or resided in high risk environment (e.g. prisons or refugee camps). | Database review | IGRA >2 years, and TST or IGRA <2 years. | 1364/5470 (24.9%) of IGRAs were positive. 358 started treatment with 91% completing treatment. 1371 IGRAs were performed on individuals not eligible based on country of origin but with an additional risk factor |
| The Netherlands | Public health services [(Spruijt et al. 2019)](https://pubmed.ncbi.nlm.nih.gov/31260502/) | Immigrants, not applying for asylum, from non-EU countries with a TB incidence of >50/100,000, all ages, intended stay in the Netherlands of at least six months. | Brochure by mail or at PHS registration | TST + confirmatory IGRA (2/5 centres) or IGRA alone (3/5 centres).  <12 years with normal immunity: TST + confirmatory IGRA. | 588 tests were offered, 566 completed. 51/249 IGRAs were positive and 227/317 TSTs were positive. 50 people had confirmatory IGRAs following TSTs. 101 people were diagnosed with LTBI, 3 with active TB. 49/101 accepted treatment, with 34 completing treatment. Increased risk of LTBI was seen in men, age groups 25-34 or >35 years, from countries with incidence >100/100k and with lower levels of education. |
|  | Public health services [(Spruijt et al., 2019)](https://erj.ersjournals.com/content/54/5/1900861) | Asylum seekers >12 years from countries >200/100,000 incidence | Asylum seekers eligible for chest radiography follow-up screening and living in an ASC | IGRA | 209/719 IGRAs were positive. Of the 209, 178 people were diagnosed with LTBI, 20 were lost to follow up, 4 were diagnosed with active TB and 3 had been previously treated for TB. 129/148 people starting TB treatment finished it. The coverage of LTBI screenings organised at the ASC (average 63%; minimum 50%, maximum 87%) was slightly higher and fluctuated less than the coverage of LTBI screenings organised at the PHS (average 59%; minimum 8%, maximum 96%) |
|  | Six community settings, run by public health services [(Spruijt et al. 2020)](https://pubmed.ncbi.nlm.nih.gov/32164637/) | Eritrean migrants entered within 10 years - moderate incidence (89/100,000) but high risk group in The Netherlands. | Invitation through mail/social media, face-to-face via community key figures, Dutch language classes, group housing, sports clubs, Eritrean church. | IGRA | 401 attended the education events. 272 presented for screening and 257 received IGRAs. 33/257 IGRAs were positive, with 29 people diagnosed with LTBI and 3 with active TB. 28/29 people completed a full course of treatment. |
