# Supplementary material for: The impact of latent tuberculosis screening programmes for migrant populations in high income, low burden countries
Source: PLoS One. 2025 Nov 13;20(11):e0335904. doi: 10.1371/journal.pone.0335904 (PMC12614610; doi:10.1371/journal.pone.0335904)
Supplement: S3 Table — (DOCX) [file pone.0335904.s003.docx]

*S3 Table: Summary of identified barriers and facilitators.*

| Theme | Item (facilitators in green, barriers in pink) | Count | Theme count |
| --- | --- | --- | --- |
| Client knowledge | Information provision | **4** |  |
|  | Comprehensive counselling | **1** |  |
|  | Communities having experienced previous TB interventions | **1** |  |
|  | Lack of understanding of LTBI | **7** |  |
|  | Misconceptions: BCG vaccine | **3** |  |
|  | Word of mouth misinformation | **1** |  |
|  | Misconceptions: active TB clearance = no TB | **1** |  |
|  | Misconceptions: TB a low socioeconomic disease | **2** |  |
|  | Misconceptions: positive TST indicates immunity | **2** | 22 |
| Collaborators and stakeholders | Collaboration with community organisations | **6** |  |
|  | Care co-ordinated with social workers / accommodation staff | **5** |  |
|  | Insufficient engagement of staff at reception centres | **1** | 12 |
| Communication | Access to interpreters or staff with same mother tongue | **5** |  |
|  | Language | **2** | 7 |
| Costs to client | Free services | **2** |  |
|  | Funded transport | **1** |  |
|  | Financial constraints | **1** | 4 |
| Divergence of practice | Frontline staff: lack of knowledge of legalities and rights | **1** |  |
|  | Frontline staff: lack of guideline knowledge | **1** |  |
|  | Lack of clear treatment guidelines | **1** |  |
|  | Low clinical priority | **2** |  |
|  | Lack of clear information about screening | **1** |  |
|  | Clinician hesitancy to treat shorter staying clients or those without permanent visas | **2** | 8 |
| Ease of access | Appointment convenience: time and place | **3** |  |
|  | Logistical: transport | **1** | 4 |
| Health system constraints | Cohesive and streamlined services | **4** |  |
|  | Fragmented services | **4** |  |
|  | Data gaps and sub-optimal data sharing between organisations | **3** |  |
|  | Lack of capacity, resources or training | **4** |  |
|  | Treatment or screening delays | **3** | 18 |
| Migrant pop. group related factors | Certain migrant population groups harder to engage | **2** |  |
|  | Low/no/delayed registration with primary care | **2** |  |
|  | Temporary accommodation | **3** | 7 |
| Social, cultural and political factors | Social risk factors e.g. homelessness, IVDU harder to engage | **1** |  |
|  | TB stigma concerns | **3** |  |
|  | Distrust of healthcare services | **4** |  |
|  | Sensitivity to being a targeted population | **1** |  |
|  | LTBI as a low priority for policy making | **1** | 10 |
| Service design | Integrated service: one-stop-shop approach or integrated with wider medical care services | **9** |  |
|  | Whole-family or friends approach | **3** |  |
|  | Service delivery in community settings | **4** |  |
|  | Testing privacy | **1** | 17 |
| Treatment | Ease of treatment: shorter treatment option or supported through dosette boxes and reminders | **3** |  |
|  | Risk based treatment selection | **1** | 4 |
|  | Pre-existing health conditions | **1** | 1 |
