# Supplementary material for: The impact of latent tuberculosis screening programmes for migrant populations in high income, low burden countries
Source: PLoS One. 2025 Nov 13;20(11):e0335904. doi: 10.1371/journal.pone.0335904 (PMC12614610; doi:10.1371/journal.pone.0335904)
Supplement: Attachment — Submitted filename: Response to reviewers.docx [file pone.0335904.s005.docx]

Friday, 21 October 2022

Dear Dr. Kawatsu and the PLOS ONE Editorial Team,

**Re: The impact of latent tuberculosis screening programmes for migrant populations in high income, low burden countries. (PONE-D-25-15733)**

I write on behalf of my co-authors to thank the reviewers for their careful review, and appreciate the opportunity to respond with a revised submission.

Please see point-by-point responses below.

Yours Sincerely,

Alice Taylor

(On behalf of the authors)

**Editor comments**

**Comment #1.1:** Please ensure that your manuscript meets PLOS ONE's style requirements, including those for file naming.

**Author’s Response:** Thank you. We have carefully reviewed the requirements and confirm we have complied the with style requirements throughout.

**Comment #1.2:** Thank you for stating the following financial disclosure:

[PM is funded by Wellcome (304666/Z/23/Z). For the purpose of open access, the author has applied a CC BY public copyright licence to any Author Accepted Manuscript version arising from this submission].

Please state what role the funders took in the study. If the funders had no role, please state: "The funders had no role in study design, data collection and analysis, decision to publish, or preparation of the manuscript."

If this statement is not correct you must amend it as needed.

Please include this amended Role of Funder statement in your cover letter; we will change the online submission form on your behalf.

**Author’s Response:** We have submitted an amended cover letter, which now includes the statement "The funders had no role in study design, data collection and analysis, decision to publish, or preparation of the manuscript.".

**Comment #1.3:** Please review your reference list to ensure that it is complete and correct. If you have cited papers that have been retracted, please include the rationale for doing so in the manuscript text, or remove these references and replace them with relevant current references. Any changes to the reference list should be mentioned in the rebuttal letter that accompanies your revised manuscript. If you need to cite a retracted article, indicate the article’s retracted status in the References list and also include a citation and full reference for the retraction notice.

**Author’s Response:** We have reviewed the reference list to ensure that it is completer and correct. New references have been added in accordance with the additions to the manuscript based on Reviewers’ comments. Reference numbers 74, 75, 76 and 77 are new additions

Lines: 658-664:

74.	Sterling TR, Villarino ME, Borisov AS, Shang N, Gordin F, Bliven-Sizemore E, et al. Three Months of Rifapentine and Isoniazid for Latent Tuberculosis Infection. N Engl J Med. 2011 Dec 8;365(23):2155–66.

75.	Huaman MA, Sterling TR. Treatment of Latent Tuberculosis Infection—An Update. Clinics in Chest Medicine. 2019 Dec;40(4):839–48.

76.	World Health Organization. Rapid communication: TB antigen-based skin tests for the diagnosis of TB infection. Geneva; 2022.

77.	Migliori GB, Dowdy D, Denholm JT, D’Ambrosio L, Centis R. The path to tuberculosis elimination: a renewed vision. Eur Respir J. 2023 Jun;61(6):2300499.

**Reviewer 1**

**Comment #1.1:** The author should discuss if the definition of "TB high endemic countries" with 150/100,000 pop. new TB cases per year is sound or if the threshold should be lower?

**Author’s Response:** Thank you.

We have added the following sentences to the limitations paragraph in the discussion:

Lines: 426-430:

Thank you.

We have added the following lines to the limitations paragraph in the discussion:

“When developing a TB screening programme, these individual circumstances should be considered. Widely recognised TB incidence thresholds (>150 per 100,000) for prioritising migrant screening programmes were used in this study. With the need for accelerated efforts to achieve TB elimination, the use of reduced incidence thresholds should be considered, such as the >100 per 100,000 population for cut off for high incidence laid out in the 2023 TB elimination framework proposed by Migliori *et al*.^74^.”

**Comment #1.2:** Perhaps it should be mentioned in the end of the discussion that also TB high endemic countries could benefit from identifying people with LTB and offer these treatment.

**Author’s Response:** Thank you for raising this important point. We agree that wider screening for LTBI and treatment might be important components of future public health strategies in high TB burden countries, but also note that literature examining the impact of such a strategy on transmission is limited currently. We have therefore added the following sentence in the limitations paragraph to acknowledge the important role of LTBI screening and management in countries of any TB burden.

Lines: 430-432:

Whilst this study is focused on strategies for LTBI detection in low-burden, high income countries, LTBI screening and management is an important component of TB programmes in countries of moderate and high TB incidence.

**Comment #1.3:** The authors include only industrialized countries and I suggest they include Oman in the review. Oman is conducting a screening program for LTB and hes the advantage that participation is mandatory to receive a work visa. See for instance:

A step forward in tuberculosis elimination: implementing migrant latent tuberculosis screening and treatment in Oman.

Alyaquobi FM et al. IJID Reg. 2025 Mar 19;14(Suppl 2):100614. doi: 10.1016/j.ijregi.2025.100614.

Cost-effectiveness of IGRA/QFT-Plus for TB screening of migrants in Oman.

Al Abri S et al. Int J Infect Dis. 2020 Mar;92S:S72-S77. doi: 10.1016/j.ijid.2020.03.010.

**Author’s Response:** Criteria for inclusion in the review were (1) high income countries as defined by the World Bank Group for the 2023/24 fiscal year, (2) low TB burden countries (<10/100,000 population) as defined by the World Health Organization 2023 TB burden estimates, (3) High net in-migration in 2021 (>10,000 migrants/year) as per the World Bank Group.

Oman was not included in the study as it did not reach the net migration criteria (-16,726 in 2021).

No changes made.

**Reviewer 2**

**Comment #2.1:** Why did the authors choose the rapid review method instead of the traditional systematic review method? Screening for LTBI is important, but not as urgent as the situation with the COVID-19 pandemic. In the methods section, please describe the reason of selecting rapid review method.

**Author’s Response:** Thank you for your comment. We recognise the merits of a comprehensive systematic review over a rapid review methodology. However, systematic reviews are resource intensive, and we unfortunately did not have the resources to conduct a full systematic review. Although LTBI screening does not represent the same acute public health emergency that the COVID-19 pandemic did, there is a reverse of the previously declining TB trends in some high-income countries such as those in the UK. Given our limited resources and the importance of providing timely insights to mitigate against these concerning trends, we opted for a rapid review approach.

We have added a short justification for this methodology.

Lines: 95:

Rapid review methodologies were used to synthesise available evidence for LTBI screening programmes, selected to facilitate the provision of timely insights to inform policy.

**Comment #2.2:** It would be useful to have a convenient table showing the expected rates of TB infection positive, LTBI treatment initiation and treatment completion if LTBI screening were performed on the migrant population. In the results section, please make the table if it is possible.

**Author’s Response:** Thank you for this comment. We agree that a table to this effect would be useful, and it was considered during the production of the study. However, during the review process we encountered substantial heterogeneity between and within studies in terms of study design, diagnostic methods (initial and confirmatory tests), criteria for test positivity, and the denominators used to report rates. Additionally, variations in clinical decision-making and definitions of treatment outcomes made direct comparisons problematic. Given these inconsistencies, we concluded that a tabulated summary could risk misinterpretation or inappropriate comparisons. Therefore, we opted for a narrative synthesis to more accurately reflect the findings.

No changes made.

**Comment #2.3:** Treatment of LTBI has progressed in recent years. 3HR regimens are more common than 6H. Additionally, a new generation of TB infection diagnostic kits is being developed and now we have new tuberculosis antigen-based skin tests. Could the coming of these new technologies affect future LTBI screening strategies for migrant populations?

**Author’s Response:** Thank you for this insightful comment. The recent advancements you mention are indeed likely to play an important role in improving treatment adherence, particularly through the increased use of shorter regimens and improved diagnostic accuracy through improved tests. Although we do note that shortened regimens e.g. 3HP remain unavailable in many low TB incidence countries, including Scotland, where this research was conducted. These developments are relevant to the future screening strategies our study population and we have added a statement to discussion section to reflect

this.

Lines: 377-384:

“Incorporation of recent advancements in LTBI screening and management have the potential to enhance screening programme effectiveness. Shorter treatment regimens, such as three months of rifampicin and isoniazid (3HR) have been associated with improved adherence and completion rates compared to longer six or nine month regimens. New *Mycobacterium tuberculosis* antigen-based skin tests (TBST) have been developed which, through limited evidence, appear to provide comparable specificity and sensitivity to IGRA testing, while retaining the operational simplicity of TST. The implementation of TBST in settings currently using TST or TST followed by confirmatory IGRAs has the potential to reduce false positives and increase cost effectiveness.”

**Comment #2.4:** In conclusion, what is the quantitative impact of LTBI screening for migrants on TB elimination or to achieve the goal of End TB Strategy in low TB incidence coutries? In the discussion section, please describe your prospect.

**Author’s Response:** Thank you for this comment. We agree that understanding the potential quantitative contribution of LTBI screening among migrants to national and global TB elimination goals is important. However, with substantial study heterogeneity, it is not possible to perform a meta-analysis and conclude with a single quantitative result. We make several key recommendations in our discussion. It was unfortunately beyond the scope of the study to directly model the impact of the implementation of key messages.

No changes made.
