# Supplementary material for: The impact of latent tuberculosis screening programmes for migrant populations in high income, low burden countries
Source: PLoS One. 2025 Nov 13;20(11):e0335904. doi: 10.1371/journal.pone.0335904 (PMC12614610; doi:10.1371/journal.pone.0335904)
Supplement: Attachment — Submitted filename: Response_to_reviewers_auresp_2.docx [file pone.0335904.s006.docx]

s

30 September 2025

Dear Dr. Kawatsu and the PLOS ONE Editorial Team,

**Re: The impact of latent tuberculosis screening programmes for migrant populations in high income, low burden countries. (PONE-D-25-15733)**

I write on behalf of my co-authors to thank the reviewers for their careful review and appreciate the opportunity to respond with a revised submission.

Please see point-by-point responses below.

Yours Sincerely,

Alice Taylor

(On behalf of the authors)

**Reviewer 1**

**Comment #1.1:** In the methods section please define the Line 95-6: Rapid review methodologies ?

**Author’s Response:** We have added a sentence to briefly explain rapid review methodologies and referenced the 2024 BMJ article by Garritty *et al*. (doi: <https://doi.org/10.1136/bmj-2023-076335>). We have also re-ordered the methodology to place the paragraph explaining that there was a single reviewer for screening and data extraction. Using one reviewer, with the advice of a second where requested, rather than two, was the key difference between this and a systematic review. This re-ordering aims to highlight that key difference.

Lines 95-103: Rapid review methodologies were used to synthesise available evidence for LTBI screening programmes, in order to facilitate the provision of timely insights to inform policy. Rapid reviews are a relatively novel methodology, used to synthesise evidence in a systematic, resource efficient manner[16]. A search strategy was developed and agreed upon by the research team prior to literature search. Articles were screened at title, abstract and full text stage by a single reviewer (AT), with a second review by PM where requested. A predefined data extraction table was designed and populated in Microsoft Excel, using the key areas of study characteristics, epidemiology, economic evaluations and barriers and facilitators. AT quality assessed individual studies using the appropriate CASP checklist according to the study design[17].

**Comment #1.2:** "selected to facilitate the provision of timely insights to inform policy". Describe the criteria for selection

**Author’s Response:** In full, this sentence is “Rapid review methodologies were used to synthesise available evidence for LTBI screening programmes, selected to facilitate the provision of timely insights to inform policy.” No formal evaluation criteria were used to select the methodology which can be described here. It was deemed the most appropriate method by the research team to answer the research question in a timely manner within the constraints of the available resources. The word ‘selected’ has been removed to avoid any confusion regarding the use of formal selection criteria.

Line 96: Rapid review methodologies were used to synthesise available evidence for LTBI screening programmes, ~~selected~~ to facilitate the provision of timely insights to inform policy.

**Comment #1.3:** Line 113-14 A protocol and search strategy were developed and agreed upon by the research team prior to literature search. Provide info on. the "protocol"

**Author’s Response:** Reference to a protocol has been removed.

Line 98: A ~~protocol and~~ search strategy w~~ere~~as developed and agreed upon by the research team prior to literature search

**Comment #1.4:** I think that the autors should mention the LTB screening program in Oman: Alyaquobi FM et al. A step forward in tuberculosis elimination: implementing migrant latent tuberculosis screening and treatment in Oman..IJID Reg. 2025 Mar 19;14(Suppl 2):100614. doi: 10.1016/j.ijregi.2025.100614.

**Author’s Response:** We thank the reviewer for this comment and providing the reference to an interesting and important new latent TB screening programme. Oman was not included in the study as it did not reach the net migration criteria required for inclusion (-16,726 in 2021). Additionally, this paper was not published at the point we conducted the search (article first submitted Mar 27 2025). Including this paper in our review would involve the alteration of our search criteria and a full update of our search criteria.

Additionally, whilst this paper is important and highlights a step forward in latent TB screening, it describes a protocol for a new programme and the potential challenges and success factors associated with it. Our study focuses on the synthesising the outcomes of existing programmes to provide insights for informing policy (Lines 133-139), and therefore this study would not fit within this selection criteria.

No changes made.

**Comment #1.5:** The discussion is very long and often repetitive. 5 pages which can be reduced to 3 pages.

**Author’s Response:** Thank you for highlighting this. We have reviewed and significantly reduced the discussion to three pages to avoid repetition of points.

Lines 323 to 408. Please refer to the tracked changes document.
